# Dietary phytochemicals in breast cancer research: anticancer effects and potential utility for effective chemoprevention

**DOI:** 10.1186/s12199-018-0724-1

**Published:** 2018-08-09

**Authors:** A. Kapinova, P. Kubatka, O. Golubnitschaja, M. Kello, P. Zubor, P. Solar, M. Pec

**Affiliations:** 1 Division of Oncology, Biomedical Center Martin, Jessenius Faculty of Medicine, Comenius University in Bratislava, Malá Hora 4C, 036 01 Martin, Slovak Republic; 2 Department of Medical Biology, Jessenius Faculty of Medicine, Comenius University in Bratislava, Malá Hora 4, 036 01 Martin, Slovak Republic; 3 Radiological Clinic, Breast Cancer Research Center, Center for Integrated Oncology, Cologne-Bonn, Rheinische Friedrich-Wilhelms-Universität Bonn, Sigmund-Freud-Str 25, 53105 Bonn, Germany; 4 Faculty of Medicine, Department of Pharmacology, University of Pavol Jozef Šafárik, Trieda SNP 1, 040 11, Košice, Slovak Republic; 5 Clinic of Gynecology and Obstetrics, Jessenius Faculty of Medicine, Comenius University in Bratislava, Kollárova 2, 03601 Martin, Slovak Republic; 6 Faculty of Medicine, Department of Medical Biology, University of Pavol Jozef Šafárik, Trieda SNP 1, 040 11 Košice, Slovak Republic

**Keywords:** Phytochemicals, Plant-derived foods, Antitumor activity, Breast cancer, Chemoprevention, Preclinical studies, Predictive medicine, Targeted prevention, Environmental health

## Abstract

Cancerous tissue transformation developing usually over years or even decades of life is a highly complex process involving strong stressors damaging DNA, chronic inflammation, comprehensive interaction between relevant molecular pathways, and cellular cross-talk within the neighboring tissues. Only the minor part of all cancer cases are caused by inborn predisposition; the absolute majority carry a sporadic character based on modifiable risk factors which play a central role in cancer prevention. Amongst most promising candidates for dietary supplements are bioactive phytochemicals demonstrating strong anticancer effects. Abundant evidence has been collected for beneficial effects of flavonoids, carotenoids, phenolic acids, and organosulfur compounds affecting a number of cancer-related pathways. Phytochemicals may positively affect processes of cell signaling, cell cycle regulation, oxidative stress response, and inflammation. They can modulate non-coding RNAs, upregulate tumor suppressive miRNAs, and downregulate oncogenic miRNAs that synergically inhibits cancer cell growth and cancer stem cell self-renewal. Potential clinical utility of the phytochemicals is discussed providing examples for chemoprevention against and therapy for human breast cancer. Expert recommendations are provided in the context of preventive medicine.

## Background

Official statistics provided by the World Health Organization demonstrates annually registered over 14 million new cancer cases, over eight million cancer-related deaths, and 32.6 million people living with cancer in 2012 worldwide [1]. *Cancer* is an umbrella term for altogether over 100 various types of the disease, which in the early twenty-first century became the acknowledged leading cause of the deaths worldwide; contextually, breast cancer plays a major role with around two million new cases and a half of million pathology-related deaths registered annually worldwide [2]. Both non-modifiable (such as genetic ones) and modifiable risk factors contribute to the manifestation of cancerous lesions. Thereby, modifiable risk factors are clearly preventable such as environmental toxic and stress factors, unhealthy lifestyle including dietary habits, amongst others, which synergistically promote carcinogenesis and clinical onset of malignancies [3–5]. Cancerous tissue transformation developing usually over years or even decades of life is a highly complex process involving strong stressors damaging DNA, chronic inflammation, comprehensive interaction between relevant molecular pathways, and cellular cross-talk within the neighboring tissues [6]. Only 5 to 10 % of all types of cancer are basically caused by inborn cancer predisposition such as the so-called familial breast cancer subtype known to be related to the BRCA1 and BRCA2 mutations. In contrast, the absolute majority of all cancer types carry a sporadic character based on modifiable risk factors [7]. The acquired DNA damage is commonly induced by strong stressors such as oxidizing agents, which can be present in food, air, and water, or they can originate from shifted metabolic pathways overproducing reactive oxygen species, e.g., in case of mitochondrial dysfunction and/or dysregulation of detoxification pathways [7, 8]. Consequently, for an efficient anticancer protection, it is crucial to maintain a stable balance between reactive oxygen species released and adequate response by detoxification pathways, production of oxidants vs antioxidants, in order to protect the sustainable molecular makeup: intact chromosomal and mitochondrial DNA, active transcriptome, and proteome pools [2, 7, 8]. Therefore, modifiable risk factors play a central role in cancer prevention. Contextually, it has been estimated that almost one-third part of all cancers could be avoided through appropriate dietary habits and supplements [9, 10]. Amongst most promising candidates for dietary supplements are bioactive phytochemicals demonstrating strong anticancer effects [11]. Their regular daily consumption may reduce a risk of several types of cancer: lung, colon, breast, cervix, esophagus, oral, cavity, stomach, bladder, pancreas, and ovary cancer [12]. However, the exact targeting mechanisms and responsible bio-ingredients are not yet fully understood [11].

Consequently, a lot of efforts have been made to explore the protective effects of a broad spectrum of plant-derived substances [13–17]. Abundant evidence has been collected for beneficial effects of flavonoids, carotenoids, phenolic acids, and organosulfur compounds affecting a number of cancer-related pathways and can slow down the carcinogenic process by suppressing survival and proliferation of tumor cells as well as diminish invasiveness and angiogenesis of tumors. Some of them can stimulate detoxifying carcinogens and eliminating them from the body [4, 18]. Further, phytochemicals may positively affect processes of cell signaling, cell cycle regulation, oxidative stress response, and inflammation [19]. Finally, they can modulate non-coding RNAs, upregulate tumor suppressive miRNAs, and downregulate oncogenic miRNAs that synergically inhibits cancer cell growth and cancer stem cell self-renewal [20, 21]. However, the biological activity of phytochemicals strongly depends on the dietary components which could either support or diminish the overall anticancer effects of the supplement [7, 22]. The objective of the present article is to update the knowledge in the area and to overview bioactive plant-derived substances, their anticancer-related biochemical properties, and mechanisms of the relevant processes. Potential clinical utility of the phytochemicals is discussed providing examples for chemoprevention against and therapy for the human breast cancer since the large scale epidemic as a characteristic for the early twenty-first century and an urgent need for innovative predictive, preventive, and personalized strategies have been recognized for this cancer type [2].

### Source of data

Data from the biomedical English language literature were reviewed in PubMed. Relevant studies were retrieved using the following keywords or MeSH (medical subject heading): “phytochemicals” or “plant-based functional foods” or “isolated plant compounds” or “fruits” or “vegetables” or “herbs” or “spices” and “antitumour activity” or “breast cancer” or “chemoprevention” or “therapy”. The focus was primarily on the most recent scientific publications.

## Phytochemicals–their definition and classification

The term “phytochemicals” refers to the bioactive non-nutrient compounds present in the plant-based diet. Numerous lines of evidence indicate that different phytochemicals in the synergy with a range of nutrients, vitamins, minerals, and fiber present in plant-derived foods, possess disease-preventive properties. It has been shown that phytochemicals possess anticarcinogenic and antimutagenic properties, and so, they can play an important role in the lowering of the various types of neoplasia [7, 18, 22–24]. This effect of phytochemicals can be expected if they are an integral component of regular human diet [25–28]. More than 5000 individual phytochemicals have been identified in plant-derived foods, such as fruits, vegetables, and grains. It is estimated that a large percentage of phytochemicals still remain unknown [11, 22]. Phytochemicals can be categorized depending on their chemical structure, botanical origin, biological properties, etc. Presently, there are several available specific databases on dietary phytochemicals and their health-promoting effects, including databases of agents ranked by their efficacy in chemopreventive preclinical studies [29]. Phytochemicals can be classified according to their chemical structure as phenolics, carotenoids, alkaloids, nitrogen-containing compounds, and organosulfur compounds [7, 22]. The most studied groups of phytochemicals are the carotenoids, phenolics, and organosulfur compounds.

*Carotenoids* are widely spread in foods. They are lipid-soluble compounds and provide color [30], and there are more than 750 structurally different carotenoids [31, 32]. Carotenoids, together with chlorophylls, have important roles in photosynthesis and photoprotection in plant tissues of phototrophic organisms [22, 31]. Carotenoids are able to quench singlet oxygen as well as to inactivate reactive oxygen species formed from exposure to light and air [33]. This benefit is also associated with its antioxidant activity in human health [22]. Carotenoids are generally categorized as follows–(a) vitamin A precursors that do not pigment, (b) pigments with partial vitamin A activity, (c) non-vitamin A precursors that do not pigment or pigment poorly, and (d) non-vitamin A precursors that pigment [30].

*Phenolics* are referred to as the secondary metabolites in plants [34]. *In planta*, they have various essential functions in the reproduction and growth [35]. Moreover, they act as defense mechanisms against pathogens, parasites, and predators; as attractants for pollinators and seed-dispersing animals; as allelopathic agents, UV protectants, and signal molecules in the formation of nitrogen-fixing root nodules; and as a contributor to the color of plants [22, 34]. The protective attributes of phenolics are due to the alteration of numerous cell signaling pathways involved in carcinogenesis such as cell cycle [36], apoptosis [37], or angiogenesis [38]. Phenolic compounds are generally classified as phenolic acids, flavonoids, stilbenes, coumarins, and tannins. Phenolic acids can be subdivided into two major groups––hydroxybenzoic acid and hydroxycinnamic acid derivatives, and represent approximately 1/3 of the phenolics in diet. Flavonoids are a diverse group of phenolic compounds that have been identified in plant-derived foods. They are classified as flavonols, flavones, flavanols, flavanones, anthocyanidins, and isoflavonoids and represent the remaining 2/3 of the phenolics in diet [7, 22].

*Organosulfur compounds* represent an important class of bioactive plant-derived substances with a wide range of purported health benefits. Based on several epidemiological and clinical trials, these compounds have shown to have anticancer activity through diverse action mechanisms [39–41]. Consuming organosulfur rich plant-derived foods, such as cruciferous vegetables, can be beneficial as they are able to protect these biomolecules from oxidative damage by hypochlorite [42]. Their protective effects against carcinogenesis were shown also in other studies [43, 44]. Glucosinolates are the important group of organosulfur compounds that act as natural pesticides and have shown antimicrobial and antifungal activities. In vitro studies have showed their antiproliferative effects on various tumor cell lines and that they are significantly involved in the metabolism of estrogen [45–47].

## Antitumor activities of bioactive plant-derived phytochemicals––mechanism of their action

**Fig. 1:**
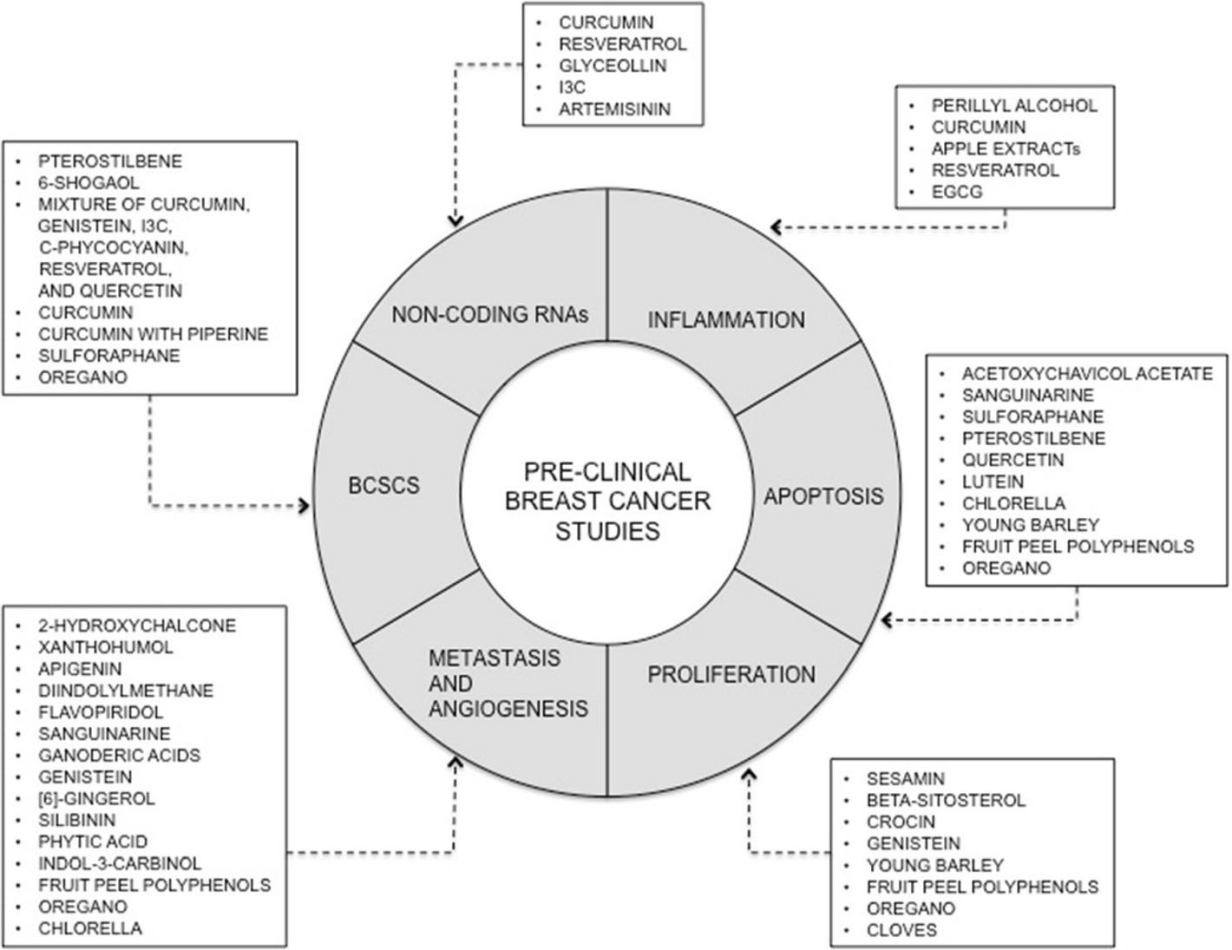
Bioactive plant-derived substances and their mechanism of action in the process of mammary carcinogenesis

Given the great structural diversity of bioactive plant-derived compounds, it is very difficult to define structure-activity relationships to deduce their underlying molecular mechanisms [18]. Therefore, a better approach is to analyze their effects on cancer-associated signaling pathways and, in this manner, define mechanisms of their action in the process of carcinogenesis. Recent results of many in vitro and in vivo studies confirming the antitumor activities of plant-derived substances clearly suggest that further research on the benefits of wide variety of phytochemicals present in whole plant-derived foods on organism is warranted (Fig. 1).

### Impact on inflammation

There is clear evidence that the immune system and inflammation play a critical role in the process of carcinogenesis and that inflammatory microenvironment is an essential component of all tumors [48]. Inflammatory responses are involved in the initiation and promotion of cancer, malignant transformation of cells, or in invasion and metastasis of cancer cells [6, 49]. Furthermore, they may affect immune surveillance and responses to anticancer therapy [6]. The immune system can eliminate premalignant and transformed cells. However, cancer cells can bypass the immune system through the growth of resistant or immunogenic clones [50]. Various immune cells are frequently found accummulated in tumors relative to the surrounding tissue. These immune cells infiltrate tumors and communicate with tumor cells [51, 52]. The important link between inflammation and carcinogenesis is the pro-inflammatory transcription factor, NF-κB [53]. Moreover, the inflammatory mediators such as pro-inflammatory cytokines stimulate also the survival and proliferation of premalignant cells and activate oncogenic transcription factors [5, 50, 54, 55]. Aharoni et al. [56] investigated the effect of polyphenols from pomegranate juice on macrophage inflammatory phenotype in vitro. In this study, polyphenols from pomegranate juice attenuated macrophage response to M1 pro-inflammatory activation in J774.A1 macrophage-like cell line in a dose-dependent manner. Dietary carotenoids (β-cryptoxanthin, astaxanthin) have the potential to affect the macrophage polarization as well [57]. On the other hand, oncogenes can initiate the inflammatory response and suppress antitumor immune response [58].

**Fig. 2:**
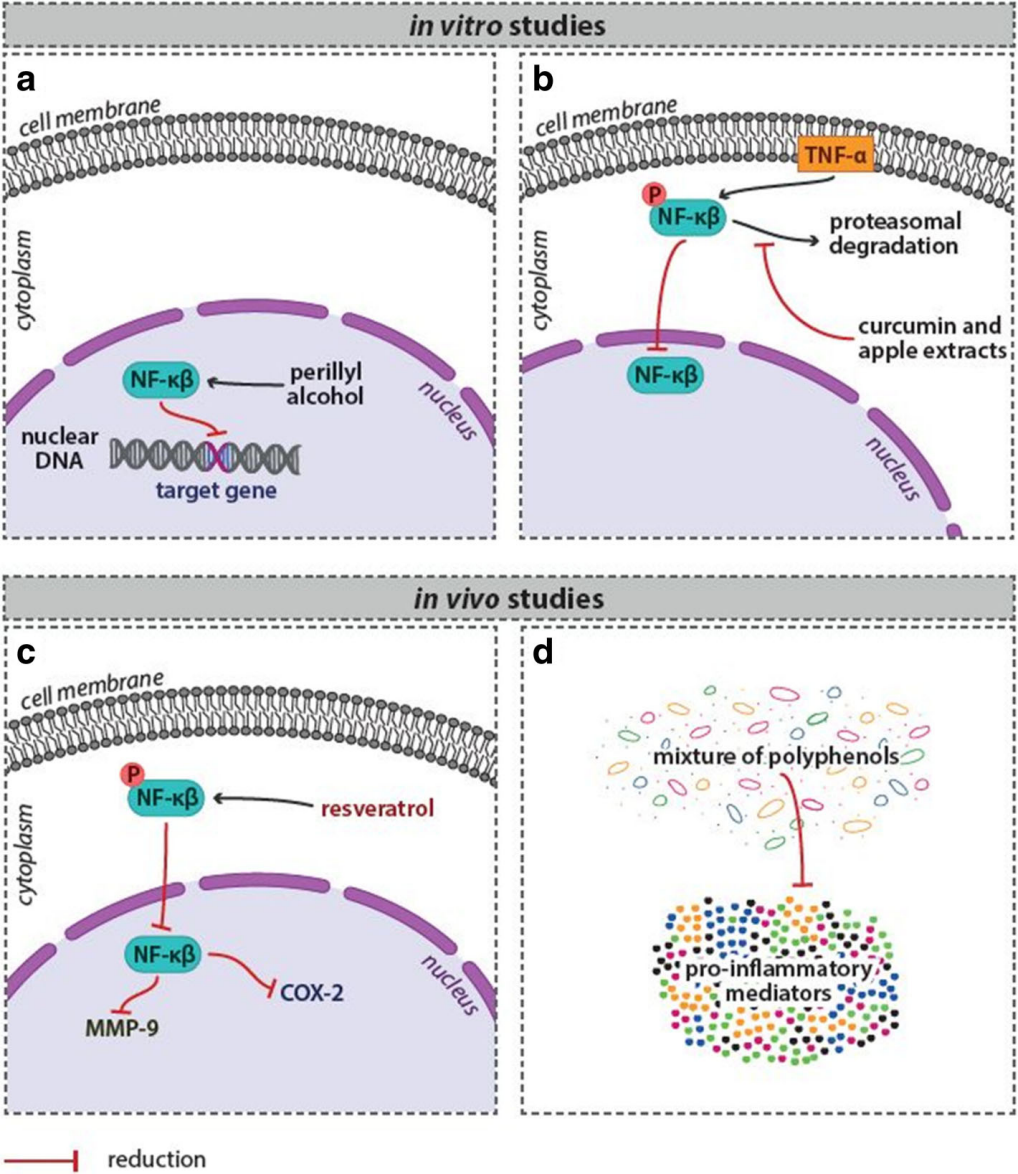
Phytochemicals reduce inflammation in breast cancer––evidence of experimental studies. For more details see the text––the section “Impact on inflammation”

Many plant-derived compounds have been found to play an important role in reducing of inflammation in breast cancer [59–65]. For example, perillyl alcohol showed impact on reduction of NF-κB DNA-binding activity and target gene induction in ER-negative mammary cells in vitro [66] (Fig. 2a). Yoon and Liu [67] showed that curcumin (at doses of 10–20 μM) and apple extracts (at dose of 5 mg/mL) significantly blocked the TNF-α-induced NF-κB activation in MCF-7 cells by inhibiting the proteasomal activities (Fig. 2b). Resveratrol used in in vivo testing reduced expression of COX-2 and MMP-9, accompanied by reduced NF-κB activation in rat breast cancer tumors [68] (Fig. 2c). In other study, Subbaramaiah et al. [69] showed that the mixture of several dietary polyphenols from Zyflamend®, including resveratrol, EGCG, and curcumin, suppressed levels of pro-inflammatory mediators (TNF-α, IL-1β, COX-2, phospho-Akt, phospho-p65, NF-κB-binding activity) in the mouse model of obesity-associated mammary gland inflammation (Fig. 2d).

### Induction of apoptosis

Apoptosis is an organized process (programmed cell death) that is continually occurring in cells [70]. Inappropriate apoptosis is a characteristic for many types of cancer. Cancer cells tend to not undergo apoptosis, allowing tumors to grow in a rapid and uncontrolled manner. The tumor suppressor gene *TP53* plays one of the most important roles in this process. Its mutation leads to functional inactivation of the p53 protein, and thus, the cell loses the important DNA damage sensor capability that normally trigger the apoptotic cascade. Some other important players in apoptosis are the cysteine proteases known as the caspases and the members of the Bcl-2 family of proteins including pro-apoptotic Bax and antiapoptotic Bcl-2.

**Fig. 3:**
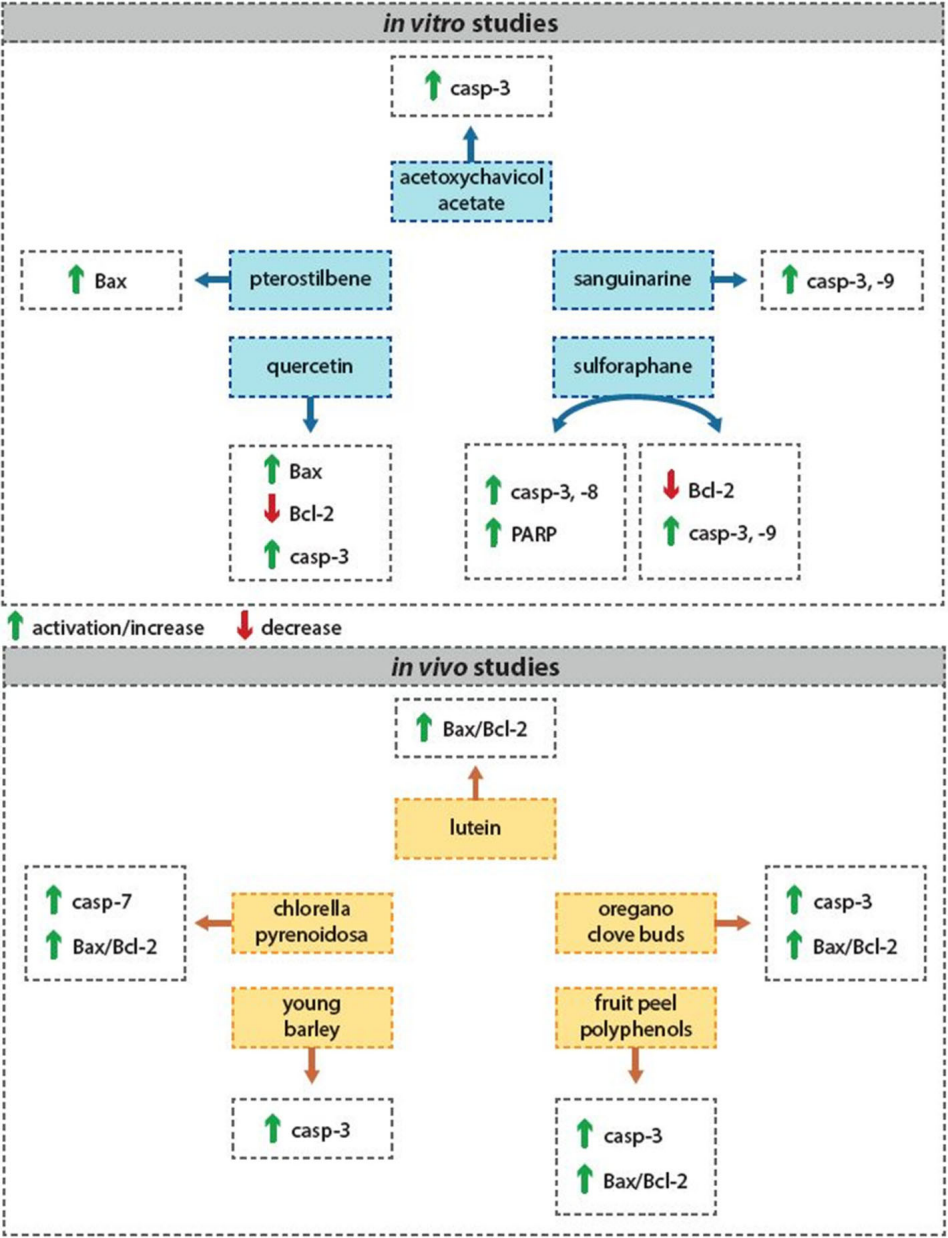
Isolated phytochemicals and/or mixture of phytochemicals contained in functional foods induce apoptosis of breast cancer cells through different mechanisms of action––evidence of experimental studies. For more details, see the text––the section “Induction of apoptosis”

Many phytochemicals or plant foods have been shown to induce apoptosis of malignant cells through different mechanisms of action. Several in vitro studies showed that the isolated phytochemicals were able to effectively trigger the activation of effector caspases in the process of apoptosis, such as caspase-3 and caspase-7, or the others, and increased Bax/Bcl-2 pro-apoptotic ratio [71–84]. There are several in vitro studies confirming these effects of phytochemicals in breast carcinoma cell lines. Campbell et al. [85] tested the effect of acetoxychavicol acetate on breast carcinoma-derived MCF-7 and MDA-MB-231 cell lines. Their results showed decrease of tumor cell viability through a caspase-3-dependent increase in apoptosis. In other study, sanguinarine induced apoptosis in MDA-MB-231 human breast carcinoma cells through several mechanisms, including activation of caspase-3 and caspase-9 [78]. A study by Pledgie et al. [86] showed that sulforaphane induced cell type-specific apoptosis in various human breast cancer cell lines. In other in vitro study, the natural dietary substance pterostilbene induced apoptosis of MCF-7 and MDA-MB-231 breast cancer cells through Bax activation [87]. Khorsandi et al. [88] demonstrated that phenolic compound quercetin is able to induce apoptosis and necroptosis in MCF-7 cells (Fig. 3). There are a little in vivo studies focusing on evaluation of antitumor effects of both single phytochemicals or their mixture in the mammary carcinogenesis. Chew et al. [89] demonstrated that dietary lutein inhibits growth of mammary tumors in female BALB/c mice by regulating apoptosis. Recently, our working group led by Dr. Kubatka has realized extensive oncological research with several whole plant-derived foods in mammary carcinoma model (Table 1). The main aim of this research is the evaluation of chemopreventive effects of long-term administration of whole plant-derived foods in a well-established model of N-methyl-N-nitrosourea (NMU)-induced mammary carcinogenesis in female rats. In all animal experiments, the chemoprevention began 7 days before NMU administration and lasted until the end of the experiment, about 13 weeks after carcinogen administration. These studies demonstrated pro-apoptotic effects of *Chlorella pyrenoidosa* (CHLO), young barley (*Hordeum vulgare* L., phylloma, BAR), fruit peel polyphenols of Flavin7® (FLA), oregano (*Origanum vulgare* L., haulm, ORE), and clove buds (*Syzygium aromaticum* L., CLO). Chlorella is a rich source of various phytochemicals, especially carotenoids and polyphenols. CHLO at a dose of 30 g/kg of chow significantly increased caspase-7 expression and Bax/Bcl-2 ratio in mammary carcinoma cells in rats [13]. Young barley represents an important source of flavonoids. Immunohistochemical analysis of tumor cells in both treated groups (3 and 30 g/kg of chow) showed significant increase in caspase-3 protein expression [14]. In the next experiment, fruit peel polyphenols of Flavin7® showed significant increase in caspase-3 expression and Bax/Bcl-2 pro-apoptotic ratio in rat mammary tumor cells (30 g/kg of chow) [15]. And in the most recent experiments, lyophilized oregano haulm (3 and 30 g/kg of chow) or cloves (10 g/kg of chow), respectively, rich in phenolic compounds and terpenoids, similarly increased caspase-3 expression and Bax/Bcl-2 ratio in rat tumor cells [16, 17] (Fig. 3). These findings confirmed the results of parallel in vitro studies in which all five natural substances were able to induce the apoptosis in MCF-7 tumor cells. In these experiments, the annexin V/PI staining, caspase-7 activation, and parallel non-caspase-dependent apoptotic pathway analyses were performed to confirm their involvement in cellular changes leading to cell death of breast cancer cell line (MCF-7). These results showed that these five natural substances induce apoptosis in MCF-7 cells through significant deactivation in antiapoptotic activity of Bcl-2 and activation of mitochondrial apoptosis pathway. Moreover, our results confirmed that the decrease in cell viability of MCF-7 cells by all tested substances was associated with an increase in the fraction of cells with sub-G0/G1 DNA content which is considered a marker of apoptotic cell death [13–16]. It is known that overproduction of ROS can promote apoptosis. In our in vitro study, chlorella significantly stimulated ROS generation in MCF-7 cells. To confirm the role of ROS in chlorella-induced cell death, MCF-7 cells were pretreated with antioxidant Trolox and compared with chlorella treatment only. Results indicate that ROS can be crucial in the induction of chlorella-induced apoptosis. Trolox pretreatment caused a reduction in ROS levels and significantly rescued chlorella-induced MCF-7 cytotoxicity [13].

**Table 1:** Evaluation of anti-tumor effects of plant-derived foods/nutraceuticals in rat mammary carcinoma model

	Tumor frequency	Tumor incidence (%)	Tumor latency (days)	References
*Chlorella pyrenoidosa*				
CONT	2.88	79.20	70.74	[13]
CHLO 0.3	2.00	80.00	74.90	
CHLO 3	1.12^a^	68.00	83.18^a^	
*Hordeum vulgare*, L. phylloma				
CONT	3.12	80.00	87.50	[14]
BAR 0.3	1.96	72.00	88.28	
BAR 3	2.72	80.00	77.70	
Flavin7®				
CONT	3.40	100.00	66.64	[15]
FLA 0.3	2.44	92.00	70.91	
FLA 3	1.44^b^	76.00^c^	74.42	
*Origanum vulgare* L., haulm				
CONT	2.96	72.00	65.33	[16]
ORE 0.3	1.32^a^	40.00^d^	75.60	
ORE 3	2.36	72.00	77.78^d^	
*Syzygium aromaticum* L., glove buds				
CONT	4.20	84.00	69.33	[17]
CLO 0.1	2.20^c^	80.00	75.25	
CLO 1	1.75^e^	87.50	76.67	

Footnote: *CONT* control group, *CHLO* chlorella group, *BAR* young barley group, *FLA* flavin group (fruit peel polyphenol extract), *ORE* oregano group, and *CLO* clove buds group. Foods/nutraceuticals were administered dietary in a concentrations of 0.3 and 3 % (3, resp. 30 g/kg of the diet), with exception of cloves with dosing of 0.1 and 1 %

Significantly different, ^a^*p* < 0.02, ^b^*p* <0 .001, ^c^*p* < 0.05, ^d^p < 0.03, ^*e*^*p* < 0.01 vs CONT

### Inhibition of proliferation

**Fig. 4:**
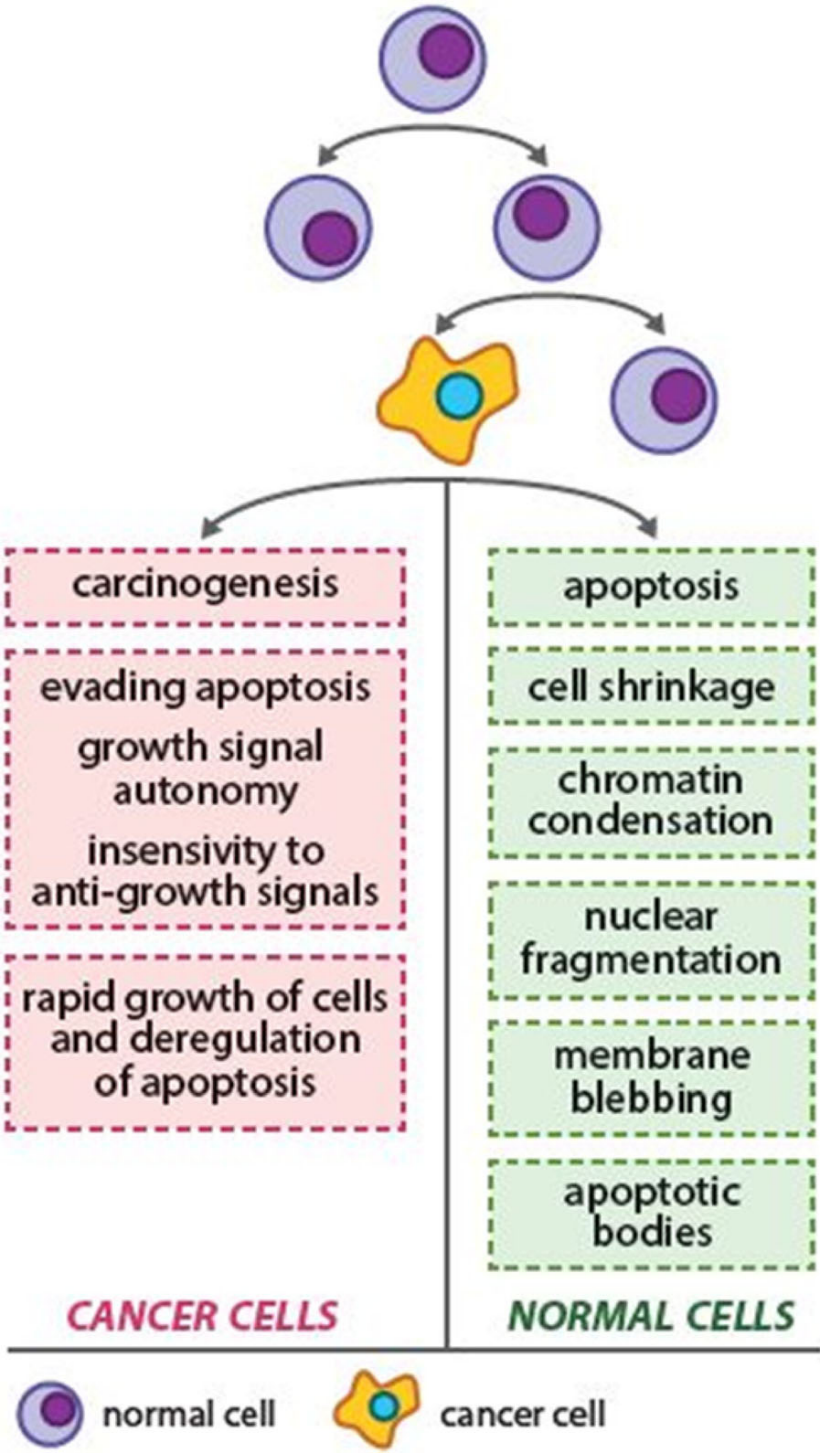
Deregulated and aggressive proliferation of cancer cells is one of the major features of carcinogenesis. For more details, see the text––the section “Inhibition of proliferation”

One of the major characteristics of carcinogenesis is a dysregulated and aggressive proliferation and rapid growth of the tumor cells. The case of normal healthy cells is their proliferation finely regulated through a balance between the growth and antigrowth signals. In this regard, apoptosis is a vital component of various processes including normal cell turnover, proper development, and functioning of many tissue/organ systems. However, cancer cells develop the ability to grow uncontrollably, and they generate their own growth signals and become insensitive to antigrowth signals [53, 90] (Fig. 4). The important factors that regulate the cell through its natural progression are the cyclins, cyclin-dependent kinases, COX-2, and c-Myc. In case of cancer, they can be upregulated causing uncontrollable cell proliferation.

In addition to pro-apoptotic effects, phytochemicals such as carotenoids, phenolic, and organosulfur compounds have demonstrated also antiproliferative effects in several in vitro studies [91–98]. They cause cell cycle arrest at various stages of the cell cycle, many of them just by affecting cyclins. Sesamin showed the ability to downregulate cyclin D1 expression in a wide variety of tumors, including human breast cancer in in vitro testing [99]. Beta-sitosterol and crocin showed inhibition of growth in human breast cancer cell lines [100, 101]. Genistein induced G2/M cell cycle arrest in MCF-7 breast cancer cell line [102]. Antiproliferative activity of another phytochemicals on breast cancer cell lines was evaluated also in further in vitro studies [103, 104]. In vitro results are commonly confronted with in vivo studies. Some animal studies showed apparent antiproliferative effects of many phytochemicals by decreasing of Ki67 expression in cancer cells [13–16, 91, 105–108]. Ki67 is considered as a good tumor marker that is present in the growing and dividing cells [109]. In our in vivo breast cancer rat model, we observed a significant decrease in the expression of Ki67 in rat mammary carcinoma cells after young barley, fruit peel polyphenols, oregano, and clove treatment. Our parallel in vitro studies confirm these results and showed the significant antiproliferative effects of these compounds in MCF-7 cell line. Chlorella, young barley, fruit peel polyphenols from Flavin7, cloves, and oregano significantly decreased metabolic activity and viability of MCF-7 breast cancer cells in MTT assay and DNA synthesis measured by BrdU proliferation assay. Moreover, these substances prevented cell cycle progression by significant decrease in G0/G1 and S populations’ enrichment [13–16].

### Impact on metastasis and angiogenesis

Cancer cell invasion and metastasis are processes which involve growth, adhesion, and migration of cancer cells, and also proteolytic degradation of tissue barriers––extracellular matrix and basement membrane [53]. Some matrix metalloproteinases (MMP-2, MMP-9) and intercellular adhesion molecule (ICAM-1) participating in the degradation of these barriers [110–113]. The important role in the process of angiogenesis and vasculogenesis plays also the receptors for vascular endothelial growth factor. Together with other growth factors such as platelet-derived growth factor, fibroblast growth factors, epidermal growth factor, and others are potentially important targets in antiangiogenic therapy for cancer. It seems that the VEGFR-2 mediates almost all of the known cellular responses to VEGF [114].

Recent reports from breast cancer studies have demonstrated antimetastatic and antiangiogenic effects of various phytochemicals. The in vitro study performed by Kim et al. [115] showed that two chalcones–2-hydroxychalcone and xanthohumol, inhibited the growth and invasiveness of triple negative breast cancer cell line MDA-MB-231. These chalcones were able to decrease the secreted level of MMP-9 in cancer cells. Way and Lin [116] showed that apigenin can play an important role in inhibition of adhesion and motility of breast cancer cells. It showed the ability to mediate the HER2-HER3-PI3K-AKT pathway in this experiment. Further, diindolylmethane decreased the CXCR4 and CXCL12 levels, in MCF-7 and MDA-MB-231 breast cancer cell lines. The chemokine receptor CXCR4 and its ligand CXCL12 are desired for metastatic activity of mammary cells [117]. In another study, flavopiridol inhibited the secretion of MMP-2 and MMP-9 in mammary cancer cells [118]. The ability to suppress invasive behavior of breast cancer cells in vitro was also by sanguinarine, ganoderic acids, genistein, [6]-gingerol, silibinin, phytic acid, and indole-3-carbinol [119–124, 47]. In some experimental studies of breast cancer, flavonoids showed the ability to inhibit the growth of mammary tumor cells by suppressing of the VEGF/VEGFR-2 signaling pathways. Mojžiš et al. [125] used the Flavin7 in in vitro testing and showed its antiangiogenic activity in HUVEC-lines. Flavin7 inhibited endothelial cell migration and capillary tube formation that indicates its potential antiangiogenic properties and also inhibited the activity of matrix metalloproteinases (MMP-9 and MMP-2) which play an important role in tumor cell invasion. In our in vivo experiment, the mixture of fruit peel polyphenols from Flavin7 significantly suppressed the VEGFR-2 expression in the treated groups of animals compared to the control [15]. Similarly, oregano (3 and 30 g/kg of chow) decreased the VEGFR-2 expression and cloves (10 g/kg) decreased the VEGF expression compared to control rat carcinoma cells in vivo [16, 17]. However, chlorella, a rich source of various carotenoids and polyphenols, showed only moderate antiangiogenic effect in our animal breast cancer model [13]. Antiangiogenic effects of phytochemicals were demonstrated in another experimental studies as well [126, 127].

### Impact on breast cancer stem cells

Cancer stem cells (CSCs), sometimes referred to as “tumor-initiating” or “tumor propagating” cells, are a small but aggressive population of cells within the tumor mass which have the ability of self-renewal, differentiation into tumor cells, invasiveness, and metastatic activity [128–130]. These rare populations of cells have been definitively identified in cancers of the hematopoietic system, brain, and breast so far. It is now believed that a cancer therapy that fails to eliminate CSCs can allow recurrence of cancer disease. Therefore, anticancer treatment strategy that specifically target CSCs should be more effective and have greater potential to reduce the risk of metastasis, multidrug resistance, or relapse in patients [131]. The subpopulation of putative human breast cancer stem cells (BCSCs) have a specific cell-surface antigen profile. Their identification from tumor samples and mammary cancer cell lines has been based mainly on CD44, CD24, and ALDH1 phenotypes. BCSCs are generally CD44 positive/CD24 negative (CD44^+^/CD24^−^) and ALDH1 positive (ALDH1^+^). Furthermore, BCSCs express higher levels of oxidative stress-responsive genes, which could be also responsible for their ability to resist anticancer therapy, than non-CSCs [128, 129, 132].

Only few in vitro or in vivo studies have evaluated the effects of plant-derived compounds (isolated or mixture) on BCSCs. Pterostilbene and 6-shogaol decreased the expression of CD44 in BCSCs in vitro. Moreover, these compounds promoted β-catenin phosphorylation through the inhibition of hedgehog/Akt/GSK3β signaling, and this way decreased the protein expression of downstream c-Myc and cyclin D1 and reduced BCSCs [133]. Ouhtit et al. [134] showed that a combination of six well-established pro-apoptotic phytochemicals (curcumin, genistein, indol-3-carbinol, c-phycocyanin, resveratrol, and quercetin) downregulated the expression of several oncostatic markers, including CD44 in MCF-7 and MDA-MB-231 breast cancer cell lines. Anti-BCSC action of curcumin (alone or in combination with piperine) was also analyzed in some others in vitro studies in breast cancer cell lines [135–137]. Reports by Li et al. [138] demonstrated that sulforaphane eliminated BCSCs in vitro and in vivo as well. This compound decreased ALDH1-positive cells in human breast cancer cell line and reduced the number and size of primary mammospheres. It eliminated also BCSCs abrogating tumor growth in mouse model. Moreover, researchers showed that sulforaphane downregulated the Wnt/β-catenin self-renewal pathway. In another study, Kubatka et al. [16] confirmed the inhibitory effect of oregano against BCSCs in rat mammary breast cancer model. The immunoexpression of CSCs markers–CD24, and EpCAM were significantly decreased in rat mammary cancer cells after oregano treatment. Using the same rat model, cloves significantly decreased CD24 and CD44 markers, however increased ALDH1 expression in mammary carcinoma cells [17]. There is a lack of date confirming anti-CSC action of phytochemicals to this date; further preclinical and clinical studies and validation of cell signaling pathways are needed in this research area.

### Modulation of non-coding RNAs

**Fig. 5:**
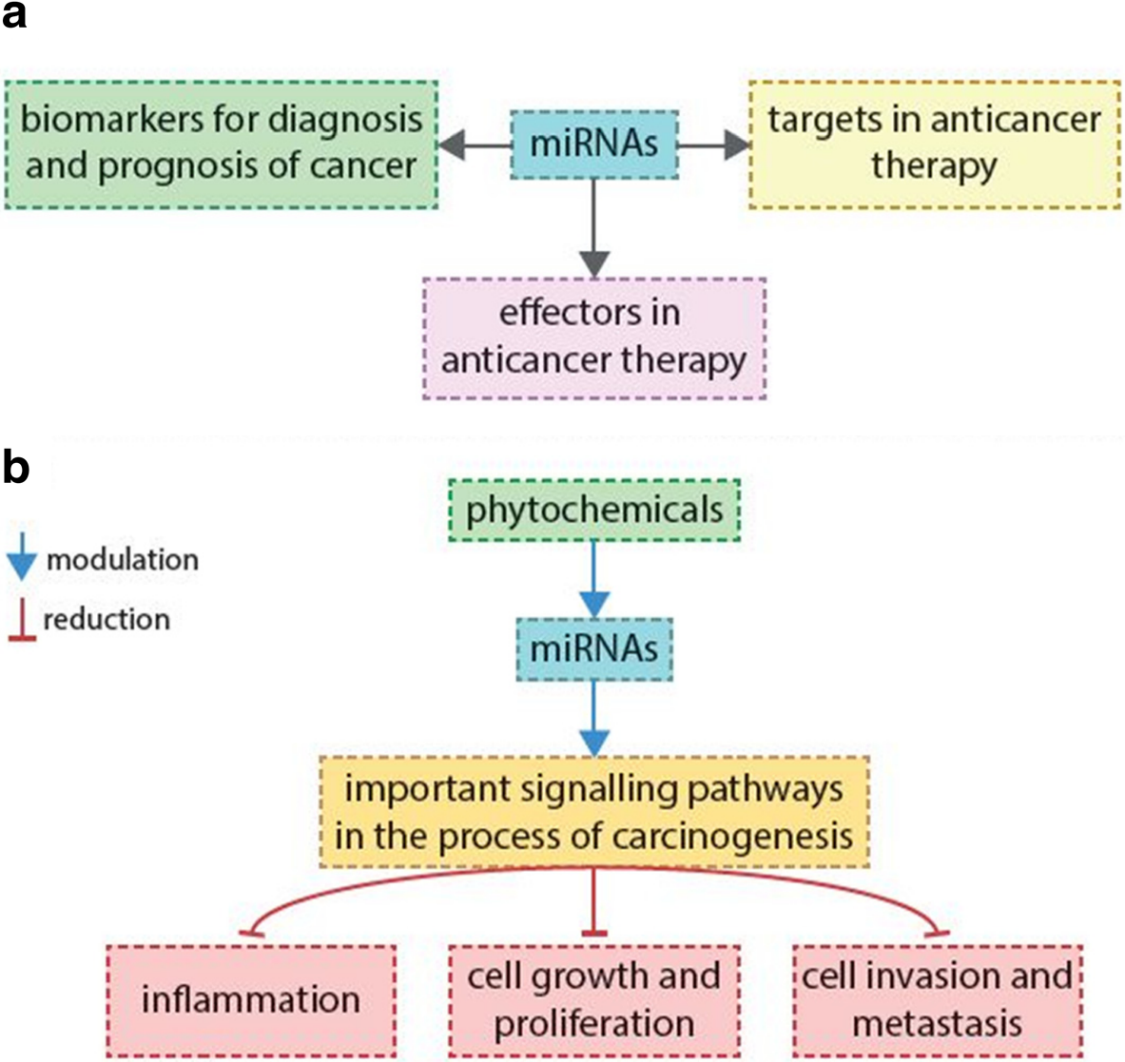
Importance of miRNAs and phytochemicals involved in the process of carcinogenesis. For more details, see the text––the section “Modulation of non-coding RNAs”

Several experimental studies dealt with modifying the activity of proteins and non-coding RNAs (ncRNAs) by plant-derived compounds. These studies have shown that phytochemicals are involved in modulating the epigenetic mechanisms and in shaping the epigenome, and thus; they could have a great importance in pharmacogenomics in the near future. It has been shown that they participate in promoter DNA methylation, histone modifications, and post-transcriptional regulation of genes through affecting ncRNAs, especially mircoRNAs and long non-coding RNAs [139]. There is growing evidence that ncRNA molecules regulate basic cellular and developmental processes both at the transcriptional and translational level under normal and cancer disease conditions [140–143]. MicroRNAs (miRNAs) are the small endogenous ncRNA molecules, often range from 20 to 22 nucleotides in length. It has been shown that they are able to regulate the gene expression through binding to the 3′ or 5′ untranslated region (3′ or 5′ UTR) of the target mRNA [140, 144]. This miRNA-mRNA interaction suppresses the expression of the target gene either through mRNA degradation or inhibition of its translation [145]. In present time, miRNAs in particular are the potential biomarkers for diagnosis and prognosis of cancer. Moreover, they can be either the potential targets for anticancer therapeutic agents or involved as effectors in the new anticancer therapeutic approaches. What is important is that one miRNA can participate in modulation of several different molecular pathways involved in the process of initiation and progression of cancer and only slight change in its expression can trigger various responses in cancer cells. Recent studies have provided convincing evidence that dietary phytochemicals are able to influence the expression of several different miRNAs in positive manner [146]. It means these miRNAs in turn modulate important cellular processes included in tumorigenesis and lead to reducing of inflammation, cell growth and proliferation, cell invasion, and metastasis. It has been shown that the miRNAs are capable both to suppress and promote oncogenesis (Fig. 5). Deregulated miRNA transcription leads to upregulation of oncogenes and silencing of tumor-suppressor genes in lung, breast, head, neck, and bone cancers [147–150]. Moreover, they can promote epithelial–mesenchymal transition (EMT) [20, 151]. However, more preclinical and clinical studies are needed to elucidate how natural compounds influence the process of carcinogenesis by influencing the levels of miRNAs.

Recent reports of several preclinical studies demonstrated that plant-derived compounds, including curcumin, resveratrol, diindollylmethane, epigallocatechin gallate, and indole-3-carbinol can alter the specific miRNAs expression, and in this way, they can increase the sensitivity of cancer cells to conventional anticancer treatment in a variety of cancer diseases [143, 152–156]. In the case of breast cancer research, resveratrol upregulated tumor-suppressive miRNAs (such as miR-16, miR-141, miR-143, and the others) in MDA-MB-231 breast cancer cell line and showed anticancer effects against BCSCs [157]. In another study, curcumin generated a miR-15- and miR-16-mediated downregulation of Bcl-2-induced apoptosis in MCF-7, Bcap-37, and SKBR-3 breast cancer cell lines [158]. Rhodes et al. [159] evaluated the anticancer activity of glyceollin belonging to the group of soy phytoalexins*.* In this in vitro study MDA-MB231 cells treated by glyceollin demonstrated a significant increase in the expression of selected miRNAs involved in EMT (such as miR-22, miR-29b, miR-29c, miR-30d, miR-34a, and miR-195) and those which act as tumor suppressors (miR-181c and miR-181d). Moreover, the significant decrease in the expression of miRNAs which are able to promote the process of carcinogenesis (miR-21) and metastasis (miR-185 and miR-224) was confirmed in this study. Results of another in vitro study in breast cancer cells performed by Hargraves et al. [160] suggest that tumor suppressive microRNA miR-34a, transcriptionally regulated by p53, is an essential component of the antiproliferative activities of some phytochemicals derived from cruciferous vegetables (I3C) and the sweet wormwood plant (artemisinin). The anticancer effects of other phytochemicals in the context of affecting the levels of miRNAs were showed in other in vitro studies as well [161–164]. Despite numerous in vitro studies dealing with effects of plant-derived compounds on levels of miRNAs in breast cancer cell lines, there is a lack of in vivo studies validating these findings. Therefore, well-designed animal studies are highly desirable and needed in the future.

## Epidemiological and clinical breast cancer studies

Several clinical epidemiological studies demonstrated that long-term and regular (several times a week) consumption of plant-based whole foods is linked with a risk reduction of breast cancer. Castelló et al. [165] described that the Mediterranean diet was related to a lower risk of breast cancer/overall risk for the top quartile vs the bottom quartile 0.56 (95% CI 0.40–0.79). Fruits, vegetables, legumes, oily fish, and vegetable oils significantly reduced the risk of mammary carcinogenesis, mainly in a triple-negative breast cancer subtype. Another Spanish study demonstrated that frequent consumption of extra-virgin oil within a Mediterranean diet provides the primary prevention of breast cancer in high-risk women [166]. Authors described that after a median follow-up of 4.8 years, 35 incident cases of breast cancer were identified. They found the rates (per 1000 person-years) of 1.1 for group with the Mediterranean diet plus extra-virgin olive oil, 1.8 for the group with Mediterranean diet plus nuts, and 2.9 for the control group. Another, Iranian case-control study included 100 incident breast cancer cases and 175 healthy controls. The results demonstrated that increased energy intakes from phytochemical-rich foods may be related to decrease the risk of breast cancer [167]. Also, blueberries and peaches demonstrated a reduction in the incidence of ER^−^ breast cancer in post-menopausal women. The multivariate relative risk for every 2 servings/week consumption for total berries was 0.82 (95% CI = 0.71–0.96, *p* = 0.01), and the relative risk for women who consumed at least one serving of blueberries a week was 0.69 (95% CI = 0.50–0.95, *p* = 0.02) compared with non-consumers. Moreover, the relative risk for consuming at least 2 servings of peaches/nectarines per week was 0.59 (95% CI = 0.37–0.93, *p* = 0.02) [168]. Most recent systematic review evaluated the association between dietary patterns and breast cancer risk. Vegetables were consistently found to be protective in breast cancer [169]. A population-based case-control study, including 2135 breast cancer cases (1070 Hispanics, 493 African Americans, and 572 non-Hispanic Whites) and 2571 controls (1391 Hispanics, 557 African Americans, and 623 non-Hispanic Whites) assessed the association between high dietary fiber intake breast cancer risk. Breast cancer risk was reduced with the consumption of bean fiber (*p* trend = 0.01), total beans (*p* trend = 0.03), or total grains (*p* trend = 0.05). Inverse associations were strongest for ER^−^/PR^−^ breast cancer [170]. Meta-analysis of He et al. [171] indicated a borderline inverse association between pre-diagnostic intake of fruit and overall survival of breast cancer, whereas intake of vegetables was not associated with survival.

There are also several clinical data about the treatment efficacy of nutraceuticals against breast cancer. In early phase clinical trials, the traditional Chinese herb *Scutellaria barbata* has shown promising efficacy and safety in patients with advanced breast cancer [172, 173]. In another clinical study, ten Chinese herbs (*Cervus Nippon Temminck*, *Ginger Charcoal*, *Citri Reticulatae Pericarpium Viride*, *Phytolaccae Radix*, *Licorice*, *Trichosanthes Kirilowii Maxim*, *Citri Reticulatae Folium*, *Panax Notoginseng*, *Epimedium Herb*, and *Fritillariae Thunbergii Bulbus*) were significantly associated with longer survival time of patients suffering from metastatic breast cancer [174].

There are limited data describing anticancer effects of isolated phytochemicals against breast cancer. Chen et al. [175] using the meta-analysis of epidemiologic studies (16 prospective and 26 case-control studies) revealed that daily dietary folate intake between 153 and 400 μg demonstrated a significant reduction of breast cancer risk in comparison with participants < 153 μg of folate intake. This effect was observed especially in women with higher alcohol intake. On the other hand, another meta-analysis of randomized trials summarized that the treatment with folic acid was not associated with the total cancer risk reduction in several cancer types including breast cancer [176]. In the meta-analysis of Hui et al. [177] (12 studies enrolling 191.419 participants), the intake of flavonols, flavones, or flavan-3-ols was associated with breast cancer reduction in post-menopausal women. Zhang et al. [178] demonstrated that epigallocatechin-3-gallate could enhance the effect of conventional anticancer therapy of breast cancer. EGCG intake decreased serum levels of VEGFs, hepatocyte growth factor, MMP-2, and MMP-9 in patients after radiotherapy.

## Discussion and future perspectives

Chemoprevention by dietary phytochemicals is an acceptable, cost-effective, and readily applicable approach to cancer control and management, but there is not sufficient evidence to show that plant-derived foods decrease the risk or prognosis of this disease. Several non-nutritive phytochemicals, either as an isolated agent or a mixture of agents from plant-derived foods, are being evaluated in preclinical and intervention trials for their potential as cancer chemopreventive agents. Despite the significant advance in our understanding of multistep process of carcinogenesis, we still know little about the mechanism of action of most chemopreventive phytochemicals. Antitumor activities of plant-derived foods are believed to be from the combination of various phytochemicals rather than an isolated agent. The complex mixture of phytochemicals with a plethora of biological activities present in whole plant-derived foods could have additive or synergistic effects against carcinogenesis. The isolated pure plant-derived compound either loses its bioactivity or may not react the same way as if the compound is present in whole foods [179].

An important challenge for research today is to identify the molecules in the cell signaling network that can be affected by individual phytochemicals for better assessment of their mechanism of action. Another important issue is the dose of phytochemicals, regarding their portion size and frequency of intake in humans. The concentrations of phytochemicals used in in vitro studies serve exclusively for one purpose, i.e., for testing the survival of cancer cells and mechanism of action of the substance; however, these doses are often unachievable in human body fluids [179, 180]. Most of in vivo studies are aimed to validate results from in vitro testing, but similarly, it is often unclear if these observations are physiologically and clinically relevant [181]. Comparing both preclinical approaches (in vitro and in vivo), animal studies could be more helpful in this respect, but another problem may be that effective dosing of phytochemicals and/or whole plant-derived foods appears to be specific among mammal species. Furthermore, pharmacokinetic properties and bioavailability of phytochemicals can play one of the key roles in investigating the dietary prevention of cancer [182]. These statements point to the fact that the use of chemopreventive compounds for interventional studies is not simple. Moreover, it is clear that preclinical oncological research provides data only for the anticancer potential of the substance.

Presently, it is generally known within scientific community that bioactive plant-derived compounds are best acquired through whole-food consumption, not from expensive dietary supplements. Phytochemicals are a low-dose component of whole plant-derived foods and are considered to be relatively non-toxic with generally positive safety profile. However, until now only a few plant-derived compounds have been scientifically proven to be safe and effective. Here, it is important to mention that phytochemicals could still display cytotoxic effects. These cytotoxic effects of phytochemicals can be due to inappropriately high-dose, unsuitable combination of drugs, or improper use. Moreover, according to the conclusions of several preclinical and clinical studies, certain phytochemicals can act as potential carcinogens or tumor promoters. For instance, beta-carotene was associated with an increased risk of lung cancer in some cancer prevention studies [183–185]. Aristolochic acids increased risk of urinary tract cancer in humans [186]. Capsaicin showed co-carcinogenic effect on TPA-promoted skin carcinogenesis in vivo [187]. Pyrrolizidine alkaloids from comfrey promoted liver carcinogenesis in rats [188]. In recent study of Johnson et al. [189], isoflavone daidzein stimulated cell proliferation of estrogen receptor positive breast cancer cells. In other study, phytoestrogens negated the effectiveness of aromatase inhibitors in estrogen-dependent breast cancer cell lines [190, 191]. In this respect, it is also necessary to be very careful in patients with hormonal or target therapy due to the possible negative interference of phytochemicals with the conventional anticancer drugs [20, 192].

Phytochemicals have great potential to improve the lives of oncological patients. Several phytochemicals are able to synergize with chemo- and radiotherapy. Therefore, their appropriate application either in the chemoprevention or potentially treatment of breast cancer would represent an attractive approach to complement conventional therapies. This appropriate combination of therapeutics could potentially lead to reduction in side effects without modifying or even increasing the therapeutic effects. For ER-positive breast tumors, one of the most used conventional therapies is selective estrogen receptor modulators such as tamoxifen and raloxifen. While these drugs are efficient for ER-positive tumors, they are not useful for ER-negative tumor subtypes. In this sense, bioactive plant-derived compounds with ability to modulate the genetic expression of tumors that are not hormone driven, could be useful [193]. Several studies have dealt with the idea of reinstating the sensitivity of ER-negative tumors by phytochemicals, and thus, these tumors could regain sensitivity to SERMs and to other anticancer drugs [194–199].

Despite the fact that epidemiologic studies has not provided sufficient evidence about anticancer activities of various phytochemicals and/or whole plant-derived foods, the results of our experimental work and other preclinical studies accentuate our confidence in the importance of regular daily consumption of whole foods full of various bioactive compounds in order to prevent or suppress the process of mammary carcinogenesis [174, 200]. The main problem of clinical research seems to be considerably weaker possibilities to control the experiment in comparison with animal or in vitro studies. Animal experiments are well controlled (strain, age, induction of carcinogenesis, diet, circadian rhytms, stressors, infections, etc.). On the other hand, in clinical trials, it is very difficult to achieve uniform conditions. From this reason, available epidemiological studies (in many cases primarily not aimed on the evaluation of anticancer effects of phytochemicals) do not validate the results. In these studies, existing positive antineoplastic effects of phytochemicals or whole plant foods could be limited by several uncontrolled risk factors in women, which are not present in laboratory conditions. Therefore, only carefully designed and controlled clinical trials can achieve significant anticancer effects of phytochemicals (or their mixtures) in humans [179]. However, it seems highly probable that phytochemicals/whole plant foods containing a high antioxidant activity (supposed genoprotective effects) may play a potentially important role cancer chemoprevention, possibly via the initiation phase of carcinogenesis.

Breast cancer research based mainly on epidemiological studies on phytosubstance or plant-derived whole food has not provided convincing anticancer effects. It deals only with the effects of general eating habits, such as Mediterranean diet, fruits, vegetable, olive oil, or fiber intake on breast cancer incidence in evaluated cohorts. On the other hand, there are well-defined animal oncological studies presenting valid and significant results. Based on preclinical data, it seems that preferring plant-based functional foods instead of single phytochemicals is a preferred approach in the cancer disease management programs. However, preclinical evaluations and well-defined and controlled clinical studies analyzing the superiority of anticancer effectivity of one over the other are needed to determine their potential use in the clinical management of breast cancer [179]. Amongst the most relevant candidate plant-based whole foods with significant anticancer activities in mammary gland in vivo should be included: chlorella [13], dark fruit peels [15], oregano [16], clove buds [17], thyme (Kubatka et al., unpublished results), rosemary [201], soy germ [202], blueberries [203, 204], blackberries [205], pomegranate [206, 207], or caraway [208]. Regarding isolated phytochemicals, only curcumin [209] and epigallocatechin-3-gallate [210] showed significant anticancer effects in animal models of breast carcinoma.

Carefully designed and mechanism-based preclinical studies, especially animal studies, can provide the important information about the potential health benefits of bioactive plant-derived substances [211, 212]. This approach is necessary before the specific phytochemicals and plant functional foods can be tested in human clinical trials. Data gained from rodent breast cancer models (e.g., with the using of herbs, spices, or dark fruit) could inspire clinical breast cancer research. Important questions of the clinical oncology include (a) type of effective phytochemicals or plant whole food, (b) appropriate clinical setting either in the chemoprevention or treatment of breast cancer, (c) dosing, (d) finding of the appropriate combination of plant substances with standard chemotherapy, and (e) target population. The big challenge for scientists today remains to develop personalized supplements composed from specific phytochemicals with proven anticancer effects for each clinical situation, which can be used in cancer prevention and/or therapy, either alone or in combination with current chemotherapy [213]. This will be possible in the case of better understanding the mechanisms of action by which dietary phytochemicals can affect human health.

## Conclusions and expert recommendations

Though, so far clinical research has not sufficiently exhibited any improvement in cancer outcomes by regular consumption of phytochemicals, comprehensive preclinical studies demonstrate significant antiinflammatory, pro-apoptotic, antiproliferative, antimetastatic, antiangiogenic, and cytotoxic for cancer stem cell effects of phytochemicals in mammary carcinoma. Consequently, well-designed clinical trials are needed to establish optimal conditions and individualized treatment algorithms for the cost-effective and readily applicable chemoprevention by dietary phytochemicals. In order to reach the goal, multi-professional expertise is essential to explore, create, and implement a comprehensive approach based on the multiomic diagnostics [214, 215], individualized patient profiling [216, 217], patient stratification by phenotyping and genotyping [218, 219], disease modelling, and machine learning [220] as well as innovative screening programs linked to the targeted preventive measures [221, 222]. Current article conforms to the principles of predictive, preventive, and personalized medicine as the medicine of the future [223].

## Abbreviations

BAR 0.3/BAR 3: Experimental group with dietary administered *Hordeum vulgare* L. (phylloma young barley) in a concentration of 0.3 and 3 %
Bax: The protein encoded in human by the *BAX* gene
Bcl-2: The family of proteins encoded in humans by the *BCL2* gene
CD24: The cluster of differentiation 24
CHLO: *Chlorella pyrenoidosa*
CHLO 0.3/CHLO 3: Experimental group with dietary administered *Chlorella pyrenoidosa* in a concentration of 0.3 and 3 %
CLO 0.1/CLO 1: Experimental group with dietary administered *Syzygium aromaticum* L., in a concentration of 0.1 and 1 %
CSCs: Cancer stem cells
EpCAM: Epithelial cellular adhesion molecule
ER: Estrogen receptor
FLAV 0.3/FLAV 3: Experimental group with dietary administered Flavin7® in concentration of 0.3 and 3%
F7: Flavin7®
Ki67: The protein encoded in humans by the *MKI67* gene
miRNA: Small non-coding RNA
ncRNAs: Non-coding RNAs
ORE 0.3/ORE 3: Experimental group with dietary administered *Origanum vulgare* L., (haulm) in a concentration of 0.3 and 3%
SERMs: Selective estrogen receptor modulators
VEGF: Vascular endothelial growth factor
VEGFR-2: Vascular endothelial growth factor receptor-2
